# Integrated Electrochemical and Computational Elucidation of Nitro Blue Tetrazolium Chloride as an Efficient Leveler for Copper Microvia Superfilling

**DOI:** 10.3390/mi16060721

**Published:** 2025-06-19

**Authors:** Dong Xing, Xiangfu Wei, Jinge Ye, Mingsong Lin, Shengchang Tang, Hui You

**Affiliations:** 1 School of Mechanical Engineering, Guangxi University, Nanning 530004, China; xdd0721@st.gxu.edu.cn (D.X.); yjg3301225@163.com (J.Y.); usmlhy@iim.ac.cn (H.Y.); 2 College of Automotive Engineering, Guangxi Transport Vocational and Technical College, Nanning 530023, China; lmsong9@126.com; 3 School of Mechanical and Automotive Engineering, Guangxi University of Science and Technology, Liuzhou 545006, China

**Keywords:** microvias filling, electroplating leveler, copper electroplating, quantum chemical calculation, molecular dynamics simulations

## Abstract

Levelers are indispensable additives for achieving void-free, bottom-up superconformal copper filling of microvias. Establishing the molecular-level correlation between leveler structure and performance is therefore essential to the continued advancement of microelectronic copper-plating technology. Herein, nitro blue tetrazolium chloride (NBT) is identified as an efficient leveler for copper microvia superfilling. A multiscale strategy—combining electrochemical measurements, X-ray photoelectron spectroscopy (XPS), density functional theory (DFT) calculations, and molecular dynamics (MD) simulations—is employed to elucidate the action mechanism of NBT and pinpoint its electroactive sites. Electrochemical tests show that NBT markedly suppresses copper deposition and, together with polyethylene glycol (PEG), effectively resists competitive adsorption by bis-(3-sulfopropyl) disulfide (SPS), thereby enhancing the microvia superfilling performance of the PEG–SPS–NBT additive system. DFT results reveal that the nitro groups and tetrazolium rings constitute the primary adsorption centers on the copper surface; the nitro groups additionally strengthen intermolecular interactions between NBT and PEG. MD simulations further confirm that NBT anchors onto the Cu(111) surface predominantly through these NO_2_ groups and the tetrazolium ring, while co-adsorbed PEG enhances the overall adsorption strength of NBT. The electroplating experiment demonstrates that NBT can act as an effective leveler for microvia superfilling. Moreover, XPS analyses further confirm the synergistic co-adsorption of NBT and PEG and verify that the NO_2_ groups and tetrazolium rings are the dominant adsorption sites of NBT. Collectively, the electroplating, XPS, electrochemical, DFT, and MD findings clarify the structure–activity relationship of NBT and provide rational guidelines for designing next-generation copper-plating levelers.

## 1. Introduction

Electrochemical copper deposition offers distinct advantages for constructing micro- and nano-scale features and is therefore indispensable in manufacturing advanced interconnects such as integrated circuits (ICs) [1,2]. For high-aspect-ratio structures—including Damascene trenches in ICs and microvias in substrates and printed-circuit boards (PCBs)—voids generated during plating remain a persistent threat to electrical performance and reliability [3,4]. Eliminating these defects has therefore become a primary objective in microvia electroplating [5,6].

Industrial practice predominantly relies on organic additive systems to locally regulate the deposition rate for achieving superfilling of microvias. In two-component systems the competitive adsorption of a suppressor and an accelerator is often sufficient for sub-100 nm Damascene features [7,8,9]; larger microvias, however, require a third component—a leveler—to suppress copper deposition at high-current-density sites (feature mouths and protrusions), thereby ensuring void-free bottom-up growth and surface planarization [10,11,12]. Among the numerous levelers reported, Janus Green B (JGB) is the most classic, as it can achieve not only superfilling in microvias [13] but also high throwing power (TP) electroplated in through-holes [14]. Additionally, previous studies have demonstrated that the azo group and the positively charged quaternary-ammonium group are the primary active functional groups in JGB [13,15].

Building on this insight, numerous researchers have designed levelers that incorporate either the azo or quaternary-ammonium group. Wang et al. employed gentian violet, which contains a positively charged quaternary-ammonium group, as a leveler for through-hole electrodeposition and demonstrated that this leveler exhibited plating performance as excellent as that of JGB [16]. However, other researchers introduced quaternary-ammonium groups with varying chain lengths onto the quinacridone skeleton to develop a novel leveler [17], and the novel leveler exhibited superior plating performance compared to JGB. Additionally, they also grafted quaternary-ammonium chains onto the Sudan I scaffold, reinforcing electrostatic adsorption while preserving the azo-naphthalene π–π interactions, thereby further enhancing plating performance [18]. Another group employed quaternary-ammonium salts of varying alkyl-chain lengths as plating levelers and further observed that these levelers delivered suppression performance superior to that of polyethylene glycol (PEG) [19]. Taken together, these studies further confirm that the positively charged quaternary-ammonium group constitutes the key active functional moiety of leveler molecules.

However, growing evidence indicates that, beyond the classic active groups—positively charged quaternary-ammonium and azo groups—auxiliary modification groups such as electronegative substituents or heteroaromatic rings can markedly reinforce leveler adsorption [20], alter the diffusion ability of molecules [21,22], and enhance synergism with other additives [23]. Dow et al. compared JGB with its derivative Diamond Black (DB), revealing significant electrochemical differences despite the two molecules differing by only a single peripheral functional substituent [24]. Similarly, Wang et al. observed that Safranine T (ST), which lacks several peripheral functional substituents present in JGB, exhibited significantly reduced through-hole throwing power compared to JGB [25], whereas Zhu et al. restored microvia superfilling by introducing hydroxyl (-OH) groups into ST [20]. Therefore, using density functional theory (DFT) calculations and molecular dynamics (MD) simulations to unravel how individual functional groups dictate adsorption energetics and electron distribution is very necessary. For example, many research groups have used DFT and MD simulations to examine how different modifying functional groups affect the leveling performance of tetrazolium derivatives, because the tetrazolium scaffold is an excellent leveler backbone: the tetrazolium ring carries an intrinsic positive charge comparable to that of quaternary-ammonium groups, while offering high structural stability and low cost. Lei et al. used DFT calculations to show that introducing N/O heteroatom substituents alters the electronic structure of tetrazolium derivatives, increases their electron activity, and thereby markedly improves microvia superfilling [26]. Similarly, combined DFT and MD studies by Wei et al. revealed that electronegative groups such as NO_2_ and I redistribute the electrostatic potential of the tetrazolium backbone, create additional chemically active sites, and strengthen intermolecular interactions, ultimately enhancing microvia filling [23]. In the same vein, Teng et al. [27] combined DFT with experiments to elucidate how various tetrazolium derivatives interact synergistically with a propylene-oxide–ethylene-oxide–propylene-oxide (PEP) triblock copolymer; highly electronegative substituents were found to promote stronger cooperation with the suppressor PEP and thus further boost superfilling performance.

**Figure 1:**
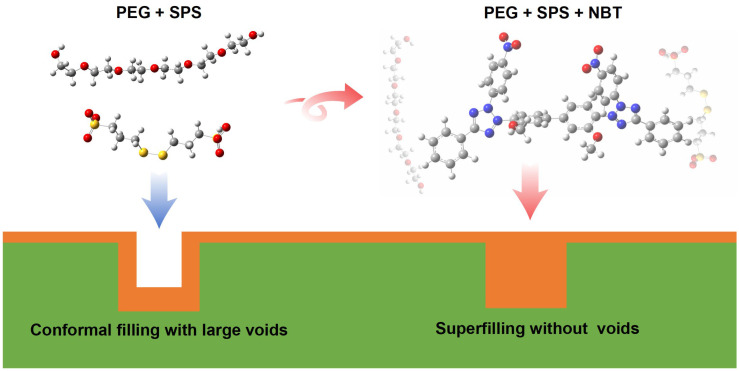
Schematic illustration of copper superfilling electrodeposition in microvias, and the chemical structure of the nitro blue tetrazolium chloride (NBT) molecule.

As is well known, nitro blue tetrazolium chloride (NBT), a commercially available tetrazolium dye, has demonstrated excellent performance as a leveler in through-hole copper filling [28,29,30]. In contrast, blind microvias present a far more challenging environment: with only one opening, electrolyte circulation is severely restricted, the diffusion layer thickens, and a pronounced ohmic and activation overpotential gradient develops. These factors make void-free filling in blind microvias significantly more difficult than in through-holes. To date, however, the suitability of NBT for blind-microvia superfilling has not been reported, nor has its structure–performance relationship been elucidated—an omission that limits the molecular-level insight into tetrazolium-based levelers and the rational design of cost-effective additives. Accordingly, NBT was chosen as the leveler in this study to assess its effectiveness in copper superfilling of microvias, of which the molecular structure is shown in Figure 1. By combining electrochemical analyses, electroplating experiments, X-ray photoelectron spectroscopy (XPS), MD simulations, and DFT calculations, we elucidate how NBT’s NO_2_ groups and tetrazolium ring govern the adsorption on Cu(111) and synergism with PEG and other species. The insights provide rational guidelines for engineering the next-generation, robust, and economical levelers.

## 2. Experimental

### 2.1. Microvia Electrodeplating and Electrochemical Measurements

Rectangular PCB fragments containing microvias with a diameter of 120 μm and a depth of 100 μm were employed as substrates for copper electrodeposition experiments. The electrodeposition experiments were performed in the basic plating solutions containing 0.88 M CuSO_4_·5H_2_O, 0.54 M H_2_SO_4_ (98%, analytical reagent), and 50 ppm HCl along with three-component system additives where NBT functioned as the leveler, PEG acted as the suppressor, and bis-(3-sulfopropyl) disulfide (SPS) served as the accelerator. The PCB substrates were electroplated at a constant current density of −10 mA/cm^2^.

Electrochemical behaviors of the additives were investigated using galvanostatic measurements (GMs) and cyclic voltammetry (CV), performed in a standard three-electrode electrochemical cell with a ParStat 4000A Electrochemical Workstation. A saturated mercurous sulfate electrode (SSE) acted as the reference electrode. GM tests were conducted under different convection conditions at a constant current density of −10 mA/cm^2^. The potential difference (Δη) of the GM curves was calculated by Equation (1):

(1) $$∆\eta =\eta_{100rpm}-\eta_{1000rpm}$$

where η_100rpm_ and η_1000rpm_ are the cathodic potentials measured at rotational speeds of 100 rpm and 1000 rpm, respectively. The CV measurements were conducted by scanning from 0.8 V to −0.8 V (vs. SSE) at a sweep rate of 20 mV/s and the rotational speed was set at 100 rpm or 1000 rpm.

A clean copper foil was immersed for 20 min in deionized water containing 30 ppm NBT, 200 ppm PEG, and 50 ppm HCl, rinsed in fresh deionized water for 5 min, vacuum-dried, and then measured by XPS. Spectra were acquired at a take-off angle of 45°, with a pass energy of 90 eV and an Al Kα excitation source (1253.6 eV).

### 2.2. Theory and Computation

All quantum-chemical calculations were carried out with Gaussian 09, using the B3LYP functional. Geometry optimizations for the isolated NBT molecule, a PEG fragment, and the NBT–Cu^2+^ complex were carried out with the def2-SVP basis set, and single-point energies were refined at the def2-TZVP level to improve accuracy. The optimized structures and electronic properties were analyzed with Multiwfn [31,32], and the graphical representations were prepared with VMD [33].

MD simulations were conducted using Materials Studio software (version 17.1) to investigate the adsorption behavior of the additives on the Cu(111) surface. The MD simulation box (46.1 Å × 44.3 Å × 77.4 Å) included nine layers of copper atoms, 1500 water molecules, additive molecules (NBT, TB, PEG, and Cl^−^), and a vacuum layer (~3 nm) at the top. The simulations, which were performed within an NVT ensemble at a temperature of 298 K using the COMPASS force field, were conducted over a total duration of 1000 ps. The adsorption energy of the additives on the Cu (111) surface (E_ads_) was calculated by Equation (2):

(2) $$E_{ads}=E_{total}-E_{Cu}-E_{additive}$$

where E_total_ is the total energy of the Cu surface with adsorbed additive molecules, E_Cu_ is the energy of the copper layer, and E_additive_ is the energy of the isolated additive.

## 3. Results and Discussion

### 3.1. Electrochemical Analysis

**Figure 2:**
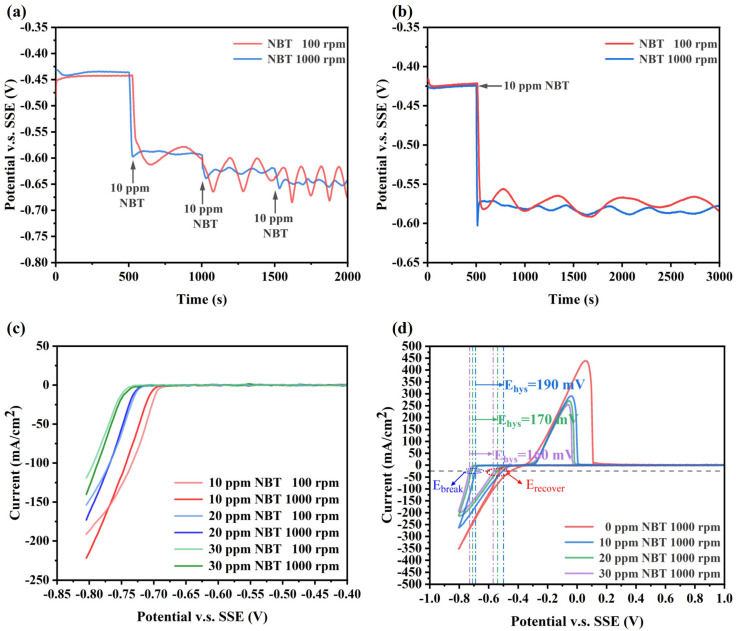
Electrochemical characterization curves of the basic plating solutions containing various concentration of NBT: (**a**,**b**) galvanostatic measurement (GM) curves obtained at a constant current density of −10 mA/cm^2^; (**c**) linear sweep voltammetry (LSV) curves; and (**d**) cyclic voltammetry (CV) curves recorded at a scan rate of 20 mV/s.

Electrochemical measurements were carried out to investigate the effect of NBT on cathodic polarization, as shown in Figure 2. From the GM curves at 1000 rpm, it can be seen that injecting just 10 ppm NBT immediately shifts the electrode potential negatively by approximately 160 mV, demonstrating that NBT strongly inhibits Cu^2+^ reduction (Figure 2a). With further increases in NBT concentration, the cathodic potential shift becomes more pronounced, indicating that NBT’s inhibition strength is concentration-dependent. Moreover, pronounced electrode-potential oscillations occur at 100 rpm but are largely attenuated at 1000 rpm, indicating that adsorbed NBT forms labile complexes with Cu^+^/Cu^2+^ whose stability is governed by Cu^2+^ mass transport rather than by NBT diffusion. As shown in Figure 2b,c, this mass-transport sensitivity appears only at lower NBT concentrations; at higher concentrations, adsorption saturates and becomes insensitive to convection. Therefore, the observed instability of the NBT–Cu complex originates from diffusion-limited Cu^2+^ depletion. Specifically, a dense NBT adlayer rapidly complexes Cu^+^/Cu^2+^ at the cathode surface, depleting the local Cu^2+^ concentration; under weak convection, insufficient replenishment forces the electrode to shift to more negative potentials to maintain the set current density. Once the overpotential reaches the threshold for NBT–Cu^+^ complex breakdown, the suppressor film collapses, releasing Cu^2+^ and causing a sudden depolarization. As the potential drifts positive beyond the collapse window, the NBT–Cu complex re-forms, re-establishing the suppressor layer and driving the potential back negative—thus generating large-amplitude, periodic oscillations. In contrast, vigorous stirring at 1000 rpm continuously supplies Cu^2+^, prevents local depletion and complex collapse, and thereby damps these oscillations. Clearly, this oscillatory behavior arises from the interplay between electrode polarization and mass transport.

The CV profiles recorded at 1000 rpm (Figure 2b) show that, in the additive-free bath, copper deposition onset occurs at −0.38 V (vs. SSE). The addition of 10 ppm NBT shifts the onset to −0.68 V, with further negative shifts at higher NBT concentrations. On the reverse scan, a pronounced hysteresis loop appears; we define its width ΔE_hys_ = E_break_ − E_recover_, where E_break_ is the forward-scan potential at −25 mA/cm^2^ onset and E_recover_ is the reverse-scan potential at the same current. Thus, ΔE_hys_ quantifies the relative ease of suppressor-film breakdown and re-formation. As NBT concentration increases, the hysteresis loop narrows, indicating that the enhanced mass-transport and adsorption capacity of NBT more rapidly rebuilds the suppressor layer [34,35,36]. This potential-dependent adsorption also explains the oscillatory polarization under weak convection: once the cathode potential becomes sufficiently negative to collapse the NBT film (E_break_), the film breakdown releases Cu^2+^ and causes depolarization, and as the potential drifts positive toward E_recover_, the NBT adlayer instantly re-forms, driving the periodic oscillations.

**Figure 3:**
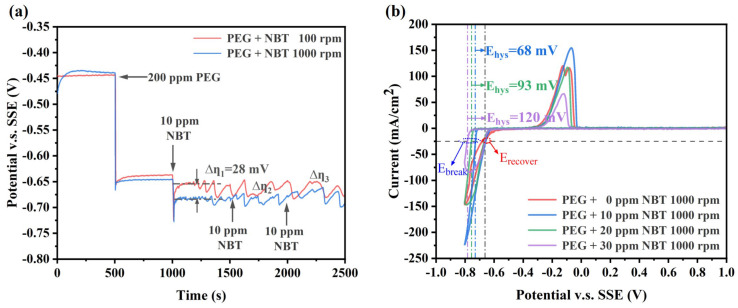
Electrochemical characterization curves of the basic plating solutions containing 200 ppm PEG and various concentrations of NBT: (**a**) galvanostatic measurement (GM) curves obtained at a constant current density of −10 mA/cm^2^; (**b**) cyclic voltammetry (CV) curves recorded at a scan rate of 20 mV/s.

To elucidate the cooperative action of NBT with the suppressor PEG, GM and CV were performed (Figure 3). The addition of 200 ppm PEG alone shifts the potential by approximately 210 mV in the negative direction (Figure 3a), reflecting formation of a dense PEG/Cl^−^ adlayer that impedes Cu^2+^ access to the cathode [37]. Upon introducing 10 ppm NBT into the PEG bath, the potential shifts an additional ∼36 mV negative at 1000 rpm, indicating synergistic co-adsorption of NBT and PEG and the formation of a more compact suppressor film (PEG–NBT) [27]. Because NBT adsorption is diffusion-limited (at low concentration), its inhibitory effect is substantially weaker under low convection (100 rpm) than high convection (1000 rpm), resulting in a large difference potential ∆η_1_ of about 30 mV and underscoring the mass-transport sensitivity of the process. Increasing the NBT concentration in the PEG bath further enhances cathodic polarization. The potential difference Δη between 100 rpm and 1000 rpm gradually narrows. Importantly, Δη remains positive, indicating that under these conditions the system transitions from NBT-diffusion control to being governed by the adsorption/desorption behavior of the PEG–NBT suppressor film. However, at high NBT concentrations, pronounced electrode-potential oscillations still occur, reflecting that the denser PEG–NBT adlayer forms stronger complexes with Cu^+^/Cu^2+^ and it remains sensitive to convection.

The CV profiles further validate the strong synergistic suppression by PEG and NBT (Figure 3b). Compared with the PEG-only baseline, the onset of copper deposition shifts progressively negative as NBT concentration increases, confirming enhanced inhibition. Unlike NBT alone (Figure 2b), the PEG–NBT curves display a large ΔE_hys_ value that largens with rising NBT concentration, while the E_recover_ remains essentially constant across all NBT concentrations—indicating the formation of a potential-sensitive PEG–NBT complex film. Higher NBT concentrations produce a denser suppressor layer that is more resistant to breakdown by electrode potential. Taken together, the PEG–NBT suppressor exhibits pronounced sensitivity to both convection and potential—properties that favor PEG–NBT-selective coverage at the microvia mouth rather than microvia bottom and promote void-free superfilling. This is due to the fact that the PEG–NBT concentration is relatively low at microvia bottom (weak convection) compared to the microvia mouth (strong convection), making the suppressor film at microvia bottom more susceptible to potential-induced collapse and thereby facilitating Cu^2+^ reduction. Conversely, at the microvia mouth the higher local PEG–NBT coverage resists breakdown and, once collapsed, rapidly re-forms, ensuring sustained suppression [38,39].

**Figure 4:**
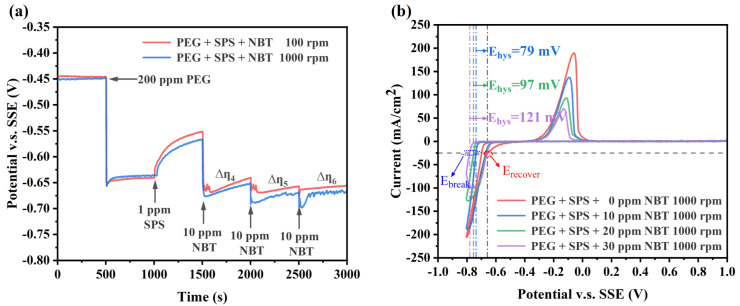
Electrochemical characterization curves of the basic plating solutions containing 200 ppm PEG, 1 ppm SPS, and various concentration of NBT: (**a**) galvanostatic measurement (GM) curves obtained at a constant current density of −10 mA/cm^2^; (**b**) cyclic voltammetry (CV) curves recorded at a scan rate of 20 mV/s.

Although NBT and PEG exhibit electrochemical behaviors that facilitate the superfilling of microvias, the achievement of efficient microvia superfilling electroplating often necessitates the synergistic effect of SPS. Specifically, SPS focuses on accelerating copper deposition at the bottom of the microvias, while PEG–NBT is responsible for suppressing copper deposition in the microvias’ entrance region. GM for the PEG–SPS–NBT additive system (Figure 4a) further confirmed the distinct yet synergistic functions of each component. After the electrode-potential drop caused by 200 ppm PEG, injection of 1 ppm SPS partially breaks through the PEG–Cl^−^ film, producing a marked depolarization [40]. Subsequent addition of 10 ppm NBT immediately reverses this trend: the potential shifts to values even more negative than those recorded with PEG alone, indicating that the PEG–NBT co-adsorbate effectively resists displacement by SPS and restores a robust suppressor layer. The differences between the depolarization curves at 100 rpm and 1000 rpm indicate that the system still exhibits a certain sensitivity to convection (∆η_4_, ∆η_5_, ∆η_6_ > 0), which is consistent with the characteristics of PEG–NBT adsorption being diffusion-limited.

The CV curves validated the observations obtained from the GM curves, as shown in Figure 4b. Relative to the PEG–SPS baseline, increasing NBT concentration again shifts the copper deposition onset to more negative potentials and produces a larger ΔE_hys_ value, while the E_recover_ remains unchanged across all NBT concentrations. This behavior indicates that higher NBT concentrations generate a denser PEG–NBT suppressor film that not only resists breakdown by applied potential but also effectively withstands SPS attack. Collectively, these electrochemical features indicate that the PEG–NBT layer remains strongly adsorbed at the microvia mouth (as discussed above in Figure 3b)—blocking SPS there—and confines accelerator action to the convection-limited via the bottom, thereby achieving void-free, bottom-up superfilling.

### 3.2. Theoretical Calculations

**Figure 5:**
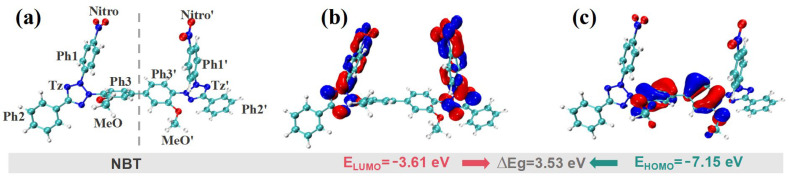
DFT calculation results for frontier molecular orbitals of the NBT molecule: (**a**) optimized molecular geometry; (**b**) lowest unoccupied molecular orbital (LUMO); (**c**) highest occupied molecular orbital (HOMO).

Quantum-chemical calculations are a powerful means of elucidating reaction mechanisms and predicting chemical reactivity. Accordingly, DFT computations were performed to analyze the electronic properties and active sites of NBT. For the frontier molecular orbitals (FMOs), the highest occupied molecular orbital (HOMO) identifies the sites most prone to donate electrons, whereas the lowest unoccupied molecular orbital (LUMO) pinpoints the sites most susceptible to accept electrons. As shown in Figure 5, the HOMO of NBT is mainly localized on the two central phenyl rings (Ph1/Ph1′) and their adjacent methoxy groups (MeO/ MeO′), indicating that these π-rich regions are the preferred electrophilic-reaction sites when NBT interacts with other species. Because the HOMO energy (−7.15 eV) is close to the Fermi level of copper (−4.5~−5 eV) [41], these sites can readily donate electron density to surface Cu orbitals, forming σ- or π-type adsorption bonds that anchor the molecule to the surface. In contrast, the LUMO is concentrated on the two tetrazolium rings (Tz/Tz′) and the nitro-substituted phenyl rings (Ph1-Nitro/Ph1′-Nitro′). LUMO-dominated sites readily accept electrons—especially from Cu 3d orbitals—thereby constituting the primary adsorption centers where feedback π-bonding occurs [42]. The dual nitro groups thus endow NBT with a strong affinity for copper. NBT displays a relatively narrow HOMO–LUMO gap (ΔE_g_ = E_LUMO_ − E_HOMO_ = 3.53 eV). Such a small gap promotes frontier-orbital overlap, facilitating charge transfer between NBT and the copper surface and, consequently, establishing a robust adsorption strength that underpins its leveling performance [43].

**Figure 6:**
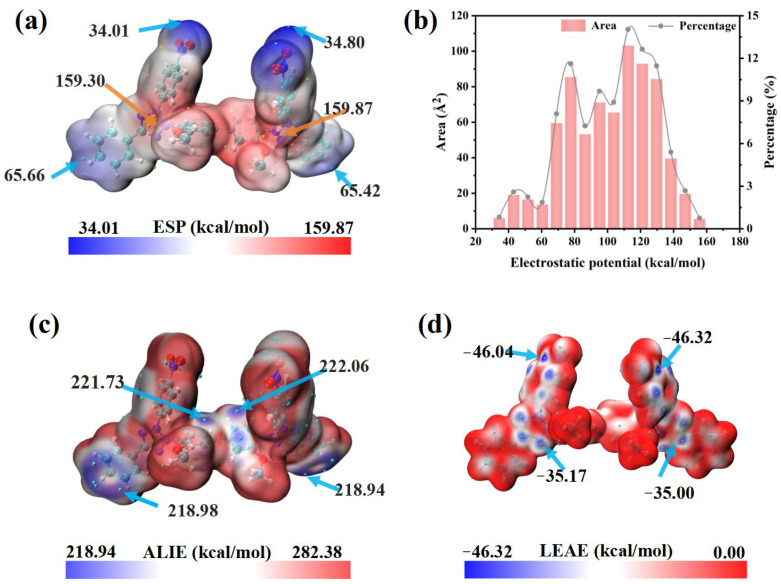
DFT calculation results for NBT molecule: (**a**) electrostatic potential (ESP) map and (**b**) corresponding histogram distribution of ESP values; (**c**) average local ionization energy (ALIE); (**d**) local electron-attachment energy (LEAE).

Molecular electrostatic potential (ESP), defined as the potential energy experienced by a unit positive test charge in the electrostatic field of a molecule, is a convenient descriptor for analyzing intermolecular electrostatic interactions. As illustrated in Figure 6a,b, the low-potential regions (blue) lie on the nitro groups (Nitro/Nitro′), whereas the high-potential regions (red) appear mainly on the tetrazolium rings themselves (Tz/Tz′) and on the methoxy-substituted phenyl rings (Ph3-Meo/Ph3′-Meo′). Because the nitro groups are strongly electronegative, its inclusion redistributes charge and makes the ESP surface more symmetric (Figure 6b), thereby improving the ability of NBT to interact electrostatically with the copper surface or with additives such as PEG [44].

The average local ionization energy (ALIE) quantifies the local propensity for electron removal within a molecule. The lowest ALIE values (≈219 kcal mol^−1^) occur on themethoxy-substituted phenyl rings (Ph3-Meo, Ph3′-Meo′) and the adjacent phenyl rings (Ph2, Ph2′) (Figure 6c), indicating that these sites are most susceptible to electrophilic attack and can donate electron density to positive species at the cathode, strengthening chemisorption [44,45]. In contrast, the local electron-attachment energy (LEAE) quantifies the ability to accept electrons; smaller values mean stronger nucleophilicity [46]. The minimum LEAE (−46.32 kcal mol^−1^) is found on the tetrazolium rings and the neighboring nitro-substituted phenyl rings (Figure 6d). This confirms that the nitro groups greatly enhance the nucleophilic reactivity of NBT, enabling efficient electron uptake from Cu 3d orbitals and the formation of robust back-donation π bonds. Such strong adsorption—especially at high-field sites such as microvia mouths—rationalizes the superior leveling performance of NBT. Moreover, this conclusion is fundamentally consistent with the findings derived from FMO.

**Figure 7:**
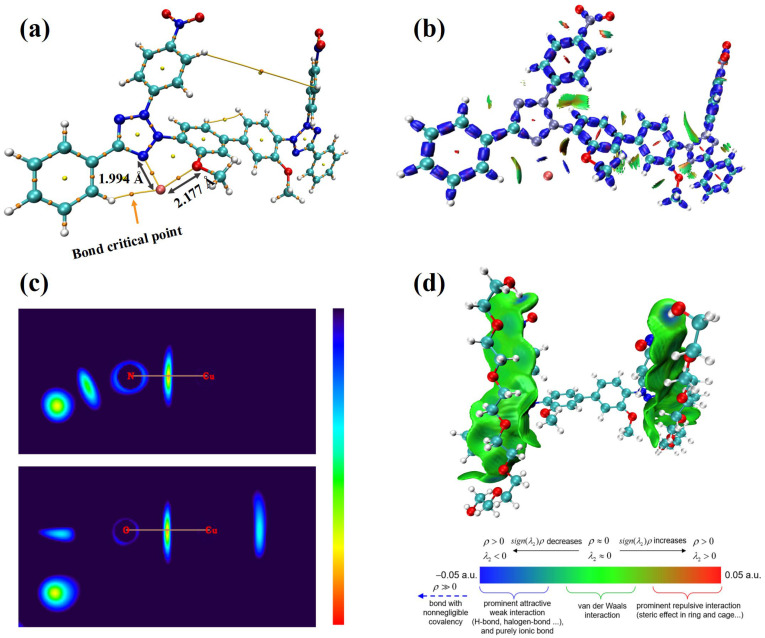
Interactions between the NBT molecule and Cu^2+^ ion and PEG fragment: (**a**) Atom in Molecules (AIM) calculation illustrating the interactions between NBT and Cu^2+^; (**b**) interaction region indicator (IRI) analysis of the NBT–Cu^2+^ interaction; (**c**) two-dimensional planar colored IRI images displaying interaction regions between NBT and Cu^2+^; (**d**) interaction between PEG and NBT visualized using the independent gradient model based on Hirshfeld partition.

To fully predict a leveler’s performance, one must assess not only its electronic active sites but also its interactions with other species and their corresponding adsorption sites. Accordingly, DFT calculations were carried out to investigate NBT’s interactions with Cu^2+^ ions and with the suppressor PEG. Atom in Molecules (AIM) and interaction region indicator (IRI) calculations were carried out for the NBT–Cu^2+^ complex, while the NBT–PEG interaction was analyzed with the independent-gradient model based on Hirshfeld partition (IGMH), as shown in Figure 7. As shown in Figure 7a, the shortest contact between Cu^2+^ and NBT is the Cu–N bond to the tetrazolium nitrogen (1.994 Å). The Cu–O distance to the methoxy group is longer (2.177 Å), indicating that the Cu–N interaction is stronger than the Cu–O one. To evaluate the bond nature, we calculated the bond-critical-point (BCP) parameters (Table 1). For both Cu–N and Cu–O bonds, the electron density at the BCP (ρ_BCP_) is below 0.10 a.u. and the Laplacian of electron density (∇^2^ρ_BCP_) value is positive, showing that the two bonds for Cu–N and Cu–O are ionic bonds [47,48]. However, only the Cu–N bond has negative energy density (H_BCP_) and negative potential energy density (Vr_BCP_), which confirms that the Cu–N bond is the stronger bond [49]. IRI analysis (Figure 7b) reveals blue isosurfaces between Cu^2+^ and the tetrazolium nitrogen, as well as between Cu^2+^ and the methoxy oxygen, indicating strong Cu–N and Cu–O interactions. The two-dimensional IRI map (Figure 7c) further supports these observations, confirming robust coordination between NBT and Cu^2+^ ions. These findings indicate that NBT coordinates predominantly with Cu^2+^ via the nitrogen atom of the tetrazolium ring, forming a strong ionic bond that retards Cu^2+^ reduction. This selective coordination promotes NBT adsorption at Cu-rich sites—such as microvia mouths or cathode protrusions—thereby efficiently inhibiting local deposition.

An effective leveler must not only coordinate with Cu^2+^ but also work synergistically with suppressors such as PEG to further enhance cathodic polarization. DFT calculations (Figure 7d) reveal that NBT–PEG interactions are dominated by van der Waals forces and a key hydrogen bond between the nitro oxygen of NBT and a hydroxyl hydrogen of PEG. The presence of this hydrogen bond drives the PEG–NBT binding energy to as high as −30.98 kcal/mol, significantly exceeding the −15.19 kcal/mol reported for the INT–PEG system (Wei et al. [23]) and the −26.47 kcal/mol for HPTT–PEP (Teng et al. [27]). The strong NBT–PEG affinity underpins the pronounced synergistic suppression observed electrochemically and underscores the pivotal role of the NO_2_ groups in both reinforcing NBT chemisorption on copper and enhancing co-adsorption with PEG, thus improving overall leveling efficiency.

**Figure 8:**
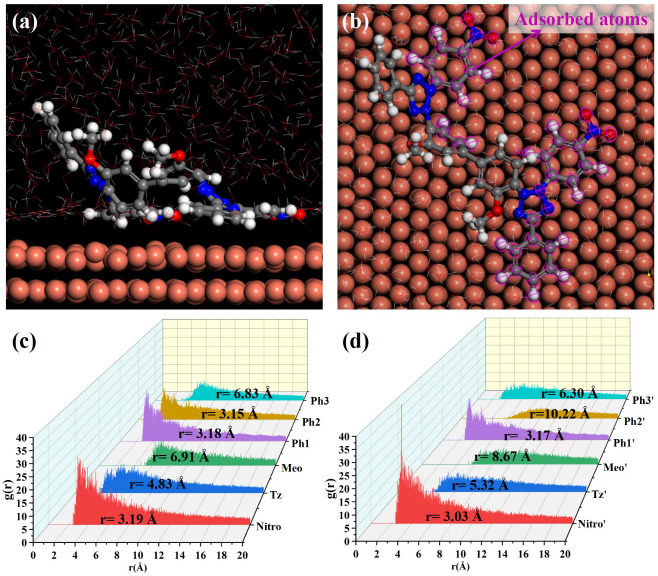
MD simulation results of the adsorption behavior of NBT molecules on the Cu(111) surface in the absence of PEG: (**a**,**b**) equilibrium adsorption configurations of NBT molecule on Cu(111) surface; (**c**,**d**) radial distribution functions (RDF) between Cu atoms on the Cu(111) surface and the centers of each NBT fragment.

MD simulations were conducted to elucidate NBT adsorption on the Cu(111) surface; the simulation results are shown in Figure 8. After equilibration without PEG, both nitro-substituted phenyl rings (Ph1-Nitro/Ph1′-Nitro′) of NBT lie flat on the Cu(111) surface, whereas the tetrazolium ring (Tz/Tz′), the methoxy-bearing phenyl ring (Ph3-Meo/Ph3′-Meo′), and the remaining phenyl ring (Ph2) stay farther from the surface (Figure 8a,b). This observation already points to the nitro groups as the primary adsorption sites.

To quantify these interactions between NBT and the Cu(111) surface, radial distribution functions (RDFs) were calculated between the centers of each NBT fragment and the surface Cu atoms, as shown in Figure 8c,d. Some studies [50,51] generally classify an adsorption peak at 1–3.5 Å as chemisorption, whereas peaks at >3.5 Å indicate physisorption. The nitro groups (Nitro/Nitro′) show peaks at 3.19 Å and 3.03 Å; the directly attached phenyl rings (Ph1/Ph1′) at 3.18 Å and 3.17 Å; and one Ph2 ring at 3.15 Å—all within the chemisorption range. By contrast, the tetrazolium rings (Tz/Tz′) exhibit broader peaks at 4.83 Å and 5.32 Å, consistent with electrostatic attraction between its positive charge and the metal. Peaks for the methoxy phenyl ring (Ph3-Meo/Ph3′-Meo′) and the remaining fragments appear beyond 6 Å, implying only weak van der Waals interaction. These results confirm that the electron-withdrawing nitro groups are the key chemisorption centers that give NBT its strong inhibitory effect, while the positively charged tetrazolium rings provide an additional electrostatic anchor.

**Figure 9:**
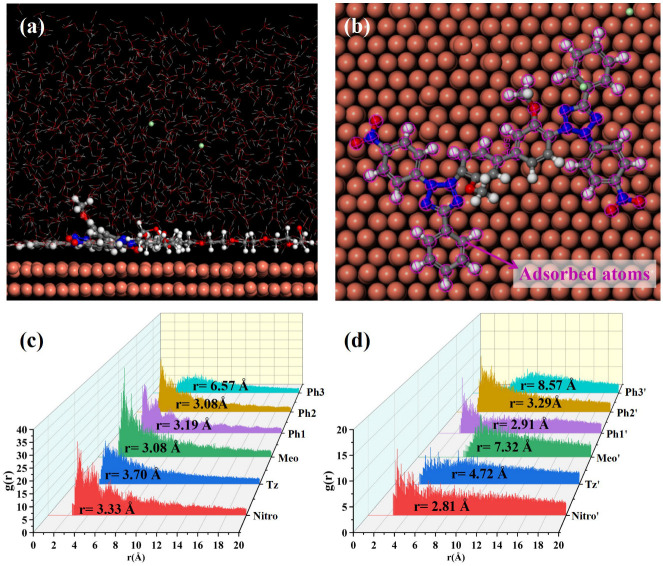
MD simulation results of the adsorption behavior of NBT molecules on the Cu(111) surface in the presence of PEG: (**a**,**b**) equilibrium adsorption configurations of NBT molecule on Cu(111) surface; (**c**,**d**) radial distribution functions (RDFs) between Cu atoms on the Cu(111) surface and the centers of each NBT fragment.

To elucidate the effect of PEG on NBT adsorption at the Cu(111) surface, MD simulations were performed on systems containing both NBT and PEG, as shown in Figure 9. Under the cooperative influence of PEG, the entire NBT molecule adopts an almost flat orientation on the Cu(111) surface, resulting in markedly tighter contact compared to the PEG-free system. The RDF analysis confirms this rearrangement: The RDF peaks for both tetrazolium rings (Tz/Tz′) shift to distances below 3.5 Å, indicating a transition from weak physisorption to strong chemisorption. By contrast, one methoxy group (Meo′) remains distant from the surface (r = 8.67 Å), and the two remaining phenyl rings (Ph3/Ph3′) only partially adsorb in a tilted orientation. Overall, PEG markedly strengthens NBT’s interfacial binding, with the calculated adsorption energy rising from −128.27 to −191.51 kcal/mol—an enhancement of nearly 50%. In short, the MD simulations demonstrate that PEG markedly enhances the chemisorption of NBT on copper, most likely through the strong NBT–PEG interaction identified by DFT. This finding is fully consistent with the synergistic suppression behavior observed in electrochemical analysis and with the DFT calculations.

### 3.3. Electrodeposition and Characterization Analysis

**Figure 10:**
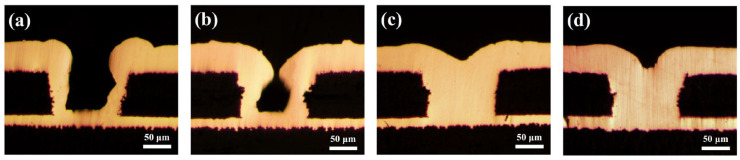
Cross-sectional optical microscope images for the microvias after being electroplated from basic plating solutions containing 200 ppm PEG, 1 ppm SPS, and various concentrations of NBT: (**a**) 10 ppm NBT, (**b**) 20 ppm NBT, (**c**) 30 ppm NBT, (**d**) 40 ppm NBT.

The study above indicates that NBT possesses the key attributes required of an electroplating leveler. To assess its practical performance, the microvia electrodeposition was carried out with 10–40 ppm NBT, and the cross-sectional results are given in Figure 10. At a low concentration of 10–20 ppm, NBT fails to support bottom-up filling: only little copper grows at the microvia’s bottom, while accelerated deposition at the mouth causes premature closure and large voids. This behavior is attributed to an insufficiently dense PEG–NBT suppressor film; its inhibition strength and potential sensitivity are too low, consistent with the narrow CV hysteresis observed in electrochemical analysis above.

However, when the NBT concentration is raised to 30–40 ppm, the filling mode switches to void-free bottom-up superfilling. The higher NBT level forms a denser, higher-potential-sensitivity PEG–NBT film that adsorbs preferentially at the relatively positive microvia’s mouth, strongly suppressing copper there. Meanwhile, SPS adsorbed at the microvia bottom—where convection is weak—accelerates copper deposition in that region, enabling void-free microvia superfilling. These plating results confirm the findings derived from electrochemical analysis, DFT calculations, and MD simulations: NBT synergizes with PEG to form a robust suppressor film that localizes at high-potential, high-convection regions, thereby enabling reliable void-free bottom-up copper filling of microvias.

**Figure 11:**
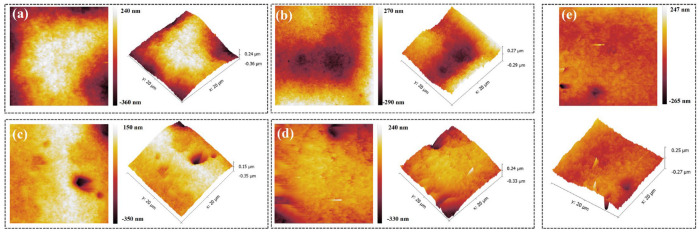
AFM plots of electroplated copper layer obtained under the basic plating solutions containing 200 PEG, 1 ppm SPS, and various concentrations of NBT: (**a**) 0 ppm NBT, (**b**) 10 ppm NBT, (**c**) 20 ppm NBT, (**d**) 30 ppm NBT, (**e**) 40 ppm NBT.

Besides filling performance, the surface-flattening ability of a leveler is a key metric. An atomic force microscope (AFM) was therefore used to examine the local morphology of the copper deposit, and the root-mean-square roughness (R_q_) and average roughness (R_a_) were quantified (Figure 11 and Table 2). With only PEG and SPS present, the deposit exhibited pronounced surface non-uniformity, with an R_q_ of 113.0 nm and an R_a_ of 131.9 nm. Introducing NBT progressively smoothed the surface: both roughness metrics declined as the NBT concentration increased, and at 40 ppm NBT the deposit displayed an R_q_ of 27.8 nm and an R_a_ of 37.2 nm. However, some isolated nodules emerged at the higher concentration of NBT, likely due to uneven NBT coverage or to local cathodic reduction of excess NBT into insoluble species that hinder normal leveling.

**Figure 12:**
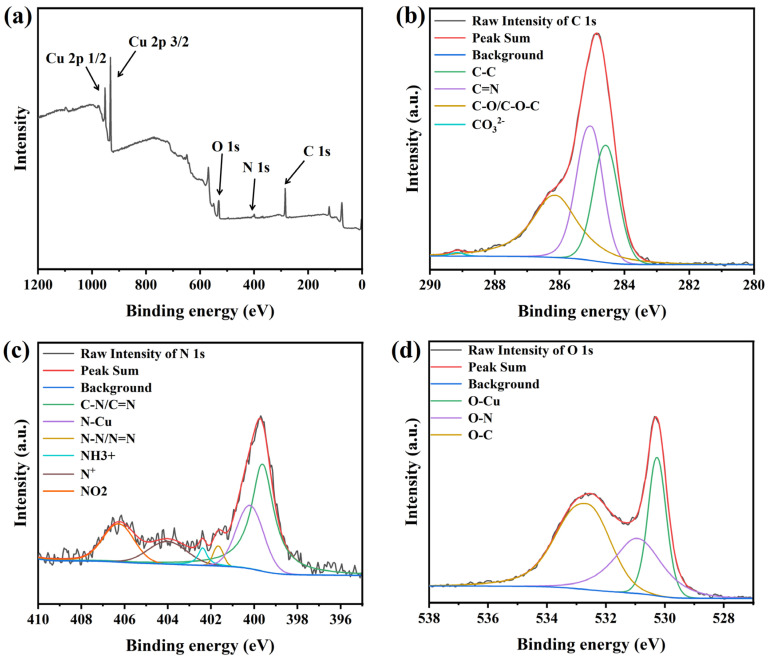
XPS deconvolution spectra of the electroplated Cu film: (**a**) full spectrum, (**b**) C 1s, (**c**) N 1s, and (**d**) O 1s.

To investigate the interactions of PEG and NBT with the copper surface, XPS was employed to analyze the elemental composition and chemical states of species adsorbed on Cu foils. Because the C, N, and O atoms in these organic additives are prone to contamination by adventitious N_2_, O_2_, and CO_2_ from air adsorbed onto the copper foil—which can obscure subtle binding-energy features—many researchers [11,17,52,53] adopt a “soak–rinse–dry” protocol to minimize interference. Accordingly, in this study the polished Cu foils were immersed for 20 min in an aqueous solution containing 200 ppm PEG and 30 ppm NBT, rinsed with deionized water, vacuum-dried, and transferred under an inert atmosphere into the XPS chamber, and the results are shown in Figure 12. The survey spectrum (Figure 12a) shows clear C 1s, O 1s, N 1s, and Cu 2p peaks, indicating that the additives are adsorbed on the copper foil surface.

The deconvoluted C 1s spectrum (Figure 12b) contains four peaks centered at 284.5 eV (C–C or C=C from PEG chains and NBT phenyl rings) [54], 285.1 eV (C–N of NBT) [55], 286.2 eV (C–O/C–O–C from ether groups in PEG or NBT) [56], and 289.2 eV (carbonate species, probably formed by trace CO_2_ uptake during immersion) [28]. These assignments indicate that both PEG and the aromatic/ether fragments of NBT are present on the surface. The N 1s signal (Figure 12c) is resolved into six peaks at 399.6 eV (C–N/C=N of NBT) [28,52,55], 400.2 eV (Cu–N, evidence of NBT binding through nitrogen) [28,57], 401.7 eV (N–N/N=N in tetrazolium) [52,55], 402.4 eV (protonated amine or quaternary nitrogen generated after interaction with Cu) [17,55], 404.1 eV (positively charged tetrazolium N^+^) [52], and 406.3 eV (nitro-group nitrogen) [52,58]. These results indicate that the tetrazolium ring and nitro groups in NBT can accept electrons from the Cu 3d orbitals and form stable coordination/back-donation π interactions with the surface. These moieties therefore act as the primary adsorption sites of NBT on copper, fully consistent with the DFT predictions. The O 1s spectrum (Figure 12d) shows peaks at 530.3 eV (Cu–O formed by interaction of ether or nitro oxygen with copper) [59], 531.0 eV (O–N from nitro and methoxy groups) [18,55], and 532.7 eV (non-bonded ether oxygen in PEG or NBT) [18,55].

Overall, the C 1s, N 1s, and O 1s spectra demonstrate adsorption of both PEG and NBT onto the copper surface. NBT utilizes its phenyl, nitro, methoxy, and tetrazolium functionalities to anchor on the Cu surface, while PEG adsorbs on the Cu surface primarily through ether oxygens and carbon backbones. These findings are in full agreement with the electrochemical analysis, DFT calculations, and MD simulations.

## 4. Conclusions

In this work, the tetrazolium derivative NBT was assessed as a leveler for copper superfilling of microvias. A combination of GM tests, CV, AFM, and XPS was applied, together with DFT calculations and MD simulations. The major findings are as follows:

(1) Electrochemical tests show that NBT alone suppresses copper deposition and shows strong synergism with PEG, forming a dense suppressor film that resists displacement by SPS. The NBT–PEG synergy is convection-sensitive and potential-sensitive, and its incorporation markedly improves the plating performance of the PEG–SPS–NBT additive system;
(2) DFT calculations identify the nitro groups and the tetrazolium ring as the principal reactive sites: both accept back-donated electrons from Cu 3d orbitals, thereby reinforcing surface adsorption. The tetrazolium nitrogens coordinate strongly with Cu^2+^ ions, and the nitro oxygens act as hydrogen-bond acceptors for PEG hydroxyls, giving a robust NBT–PEG interaction with a binding energy of −30.98 kcal/mol;
(3) MD simulations indicate that NBT alone adsorbs stably on Cu(111) through its nitro-substituted phenyl rings, with an adsorption energy of −128.27 kcal/mol. The presence of PEG increases this value to −191.51 kcal/mol and enables chemisorption via nitro, tetrazolium, and methoxy sites, an effect attributable to strong NBT–PEG intermolecular forces;
(4) Electroplating experiments confirm that 30–40 ppm NBT yields void-free bottom-up filling in microvias. XPS verifies co-adsorption of PEG and NBT; the NBT anchors to the copper surface through its nitro, tetrazolium, methoxy, and aromatic sites. These mutually consistent results clarify the structure–function relationship that underlies the effectiveness of NBT as a leveler for microvia superfilling.

## Figures and Tables

**Table 1:** The key parameters for AIM analysis.

Bond-Atoms	ρ_BCP_ × 10^2^ (a.u.)	∇^2^ρ_BCP_ × 10^2^ (a.u.)	H_BCP_ × 10^3^ (a.u.)	Vr_BCP_ × 10^2^ (a.u.)
Cu^2+^–N	8.07	50.64	−1.37	−12.93
Cu^2+^–O	4.77	26.66	2.91	−6.08

**Table 2:** Roughness parameters of copper layer electroplated under the basic plating solutions containing 200 PEG, 1 ppm SPS, and various concentrations of NBT.

NBT	0 ppm	10 ppm	20 ppm	30 ppm	40 ppm
R_q_ (nm)	113.0	87.7	53.8	56.8	26.7
R_a_ (nm)	131.9	105.1	71.0	74.5	35.7

## Data Availability

Data will be made available on request.
